# Immune Landscape of the Tumor Microenvironment Identifies Prognostic Gene Signature CD4/CD68/CSF1R in Osteosarcoma

**DOI:** 10.3389/fonc.2020.01198

**Published:** 2020-07-24

**Authors:** Yi-jiang Song, Yanyang Xu, Xiaojun Zhu, Jianchang Fu, Chuangzhong Deng, Hongmin Chen, Huaiyuan Xu, Guohui Song, Jinchang Lu, Qinglian Tang, Jin Wang

**Affiliations:** ^1^Department of Musculoskeletal Oncology, Sun Yat-sen University Cancer Center, Guangzhou, China; ^2^State Key Laboratory of Oncology in Southern China, Collaborative Innovation Center of Cancer Medicine, Guangzhou, China; ^3^Department of Pathology, Sun Yat-sen University Cancer Center, Guangzhou, China

**Keywords:** osteosarcoma, tumor immune microenvironment, prognostic gene signature, tumor infiltrating immune cells, tumor-associated macrophages, tumor-infiltrating lymphocytes

## Abstract

**Background:** Osteosarcoma (OSA), the most common primary bone malignancy in children and adolescents, is prone to metastases and unfavorable prognosis. Owing to its strong genomic heterogeneity, traditional chemotherapy, or targeted immunotherapy has not effectively improved the related overall survival for decades. Since the landscape of the OSA tumor immune microenvironment is scarcely known, despite it playing a crucial role in predicting clinical outcomes and therapeutic efficacies, we aimed to elucidate its molecular characteristics.

**Methods:** The immune signature of 101 OSA samples was explored using transcriptome profiling and clinical characteristics retrieved from the Therapeutically Applicable Research to Generate Effective Treatments (TARGET) program. Correlations between the prognostic immune markers and their clinical chemotherapy responses were assessed and verified based on 45 OSA primary tumors.

**Findings:** We identified the heterogeneity underlying tumor immune signature in OSA, and found CD4+ T cells and macrophage markers CD4/IFNGR2/CD68 to be feasible prognostic factors, exerting significantly positive correlation with each other. Specifically, CSF1R, which plays an essential role in the regulation of proliferation and differentiation of macrophages, was found to be a specific signature associated with CD4/CD68, with improved OSA clinical outcomes.

**Interpretation:** The immune landscape based on CD4/CD68/CSF1R gene signatures showed considerable promise for prognostic and therapeutic stratification in OSA patients. A specific immune signature for OSA, abundantly consisting of Th1-polarized CD4+ T cells and CSF1R-related CD68+ macrophages, may improve the predictive efficacy of chemotherapy and improve prognosis in patients with OSA.

## Introduction

Osteosarcoma (OSA) is the most common primary malignant bone tumor in children and young adults (1), and is prone to metastases and unfavorable prognosis. Our preliminary work had confirmed high-level genomic instability in OSA, as reported in previous studies (2, 3), which may impede the efficacy of targeted therapy or targeting of immune checkpoint pathways. Since PD-1 inhibition has limited activity in OSA, only a fraction of patients may benefit from therapeutic intervention (4). Thus, there is no specific, safe, and effective treatment till date capable of improving the overall survival rates of OSA patients. Hence, therapeutic strategies have remained stagnant over the past three decades (5, 6).

In recent years, studies have reported combination strategies, including chemo-immunotherapy and dual-immunotherapy, to possibly prolong the overall survival of patients (7). Immunosuppressive cancer microenvironments are now recognized as major impediments and key determinants to the efficacy of chemotherapy or checkpoint inhibitors of immunotherapy (8, 9), owing to the presence of tumor-associated macrophages (TAM) and tumor-infiltrating lymphocytes (TILs), which can inhibit immune-mediated anti-tumor effects (10). The immune signatures of cytotoxic T lymphocyte (CTL) infiltration, IFNγ secretion, and checkpoint activation have been proven to be associated with prolonged clinical outcomes, in different cancer types (11, 12). Hence, investigation of the immune landscape of tumor immune microenvironment (TIME) could help identify suitable immune biomarkers, allowing for stratification of potential therapeutic agents in OSA patients.

Macrophages, as critical regulators of tumor immunity, have the ability to directly or indirectly suppress T cell responses (13). Similarly, CD4+ T cells can directly act by eliminating tumor cells through cytolytic mechanisms, or indirectly by modulating the tumor microenvironment (14). M1- or M2-dominant macrophage responses can dictate whether CD4+ T helper (Th)-type or other types of inflammatory responses occur (15). For instance, M1 macrophages can amplify Th1 responses, providing a positive feedback loop in the anti-tumor response by recruiting large number of Th17 cells (16).

Tumors with high percentage of macrophage-colony stimulating factor (M-CSF or CSF-1) have predominantly been associated with monocyte infiltration (17, 18). Ponzetta et al. reported that anti-CSF1R antibody-treated 3-MCA-induced sarcomas and CSF1R-mediated TAM depletion drastically increased carcinogenesis in granulocyte-CSF-R competent mice while reducing tissue levels of IL-12p70 and IFNγ (19). Furthermore, Neubert et al. had examined whether CSF1/CSF1R expression correlated with the abundance of CD8+ T cells and CD68+ TAMs in melanoma, suggesting that melanoma infiltration by CD8+ T cells correlates with enrichment of CSF1^+^ and CSF1R^+^ (20). Administration of the M-CSF/CaCO_3_ nanoparticle was found to significantly inhibit tumor growth by promoting T-cell tumor infiltration and reversing the M1/M2 polarization balance of the microenvironment in a B16 melanoma model (21). Hence, CSF1R+ TAM has been proven to play an important role in immuno-oncology, and has been considered an antineoplastic immunotherapeutic target in recent years (7).

For the advancement of effective immunotherapy in precision medicine, integration of large clinico-genomic datasets and computational modeling may prove effective for deciphering resistance to immunotherapy based on the subclasses of TIME and their interplay (22). In this study, we described the immune landscape of the OSA tumor microenvironment, along with its heterogeneity, and uncovered the abundant and predominant infiltration of relevant immune cell subtypes (CD4+ Th1 cells and CD68+ macrophages) in the microenvironment.

## Materials and Methods

### Transcriptome Data Processing

The results published here are in whole or part based upon data generated by the Therapeutically Applicable Research to Generate Effective Treatments (https://ocg.cancer.gov/programs/target) initiative, phs000218. The data used for this analysis are available at https://portal.gdc.cancer.gov/projects. Level three RNA-Seq data and relevant clinical information of patients with OSA were downloaded from the TARGET OSA project (https://ocg.cancer.gov/programs/target/projects/osteosarcoma), and enrolled in our study accordingly. Of the 101 OSA RNA-Seq samples, 89 had complete prognostic information. We used TPM (Transcripts Per Kilobase Million) values and normalized them for gene expression analyses; TPM values were log_2_-transformed, using an offset of 1 in order to avoid errors.

### Differential Expression and Cluster Analysis

R/Bioconductor package *limma* was used for finding the differentially expressed genes (DEGs) with *p*-values <0.05 and fold-change >2. Enrichment analyses of DEGs were performed using Metascape (23). Clustering was performed in R using the function *hclust*. Each feature was scaled to a mean of 0 and standard deviation of 1, before hierarchical clustering analysis and heat map plotting. For heat map generation, we used the *annotation_col* arguments to group the samples before drawing in the R package *pheatmap*.

### Gene Set Enrichment Analysis

To gain further insight into biological implications, we performed gene set variation analysis (GSVA) and identified the pathway alterations that underlie our dataset (24). We also used the single-sample Gene Set Enrichment Analysis (ssGSEA) method to conduct enrichment analysis on the same input dataset (25). The ssGSEA method uses the difference in empirical cumulative distribution functions to calculate enrichment statistic per sample, and further normalizes it by the range of values taken throughout the gene sets and samples.

### Immune Infiltration

We used two analytical tools for immune infiltration in this study. First, ESTIMATE algorithm was selected to calculate stromal and immune scores and infer the overall level of infiltration of stromal and immune cells by ssGSEA (26). Next, ImmuCellAI (Immune Cell Abundance Identifier) was used to estimate the abundance of 24 immune cell subtypes from gene expression dataset (27), including 18 T-cell subtypes and 6 other immune cells including B cells, NK cells, Monocytes, Macrophages, Neutrophil cells and DC cells.

### Weighted Correlation Network Analysis

Weighted correlation network analysis (WGCNA) was used to identify modules of highly correlated immune genes, summarizing the clusters using the module eigengene or an intramodular hub gene, for relating modules to one another and to an external clinical phenotype (28).

### Survival and Regression Analysis

Survival analysis was performed to assess the effects of immune cell types and genes associated with recurrence. The Kaplan-Meier method was used to calculate survival; estimates were made with a log-rank analysis using the *survival* and *survminer* packages in R. To choose the best TPM cut-offs for grouping patients most significantly, patients were stratified according to quartiles of TPM value from the TARGET cohort. Low (25% quantile) and high expression (75% quantile) was used to group the patients, and significant differences in survival outcomes of the groups were examined.

The R function *coxph* was used for applying univariate Cox regression to genes in the Cox proportional-hazards model. Receiver Operating Characteristic (ROC) analysis was conducted using the package *pROC* in R. Before Cox regression analysis, we excluded all genes with low expression, i.e., those with a median TPM expression among samples <10.

### Patients and Tissue Samples

Primary surgical tissue samples of OSA were obtained from 45 patients (median age 15 years, range 6–44 years) at Sun Yat-sen University Cancer Centre, from November 2017 to October 2019. All patients received four to six cycles of neoadjuvant chemotherapy with MAPI regimen before the surgical resection of primary tumors. Clinical response categories of neoadjuvant chemotherapy were evaluated according to RECIST1.1 criteria (Supplementary Table 1), considering change of tumor volume based on magnetic resonance imaging (MRI) and the development of pulmonary metastasis based on chest computed tomography (CT), as reported previously (29–31). According to RECIST, progressive disease (PD), partial response (PR), and stable disease (SD) were calculated. The resected OSA tissues were collected, cut into specimens of no more than 0.5 cm, and submerged into RNAlater™ Stabilization Solution (Invitrogen™, catalog #AM7021) at 4°C overnight to stabilize and protect the RNA during surgical resection of the primary tumor; thereafter, RNAlater was removed and RNA -tissue samples were transferred and stored at −80°C.

### Samples Lysis and RNA Isolation

Fresh sample specimens containing RNA were thawed at 4°C, transferred into 2.0-mL tissue grinding tubes containing mill beads, lysed in TRIzol™ Reagent (Invitrogen™, catalog #15596018), homogenized, and centrifuged thrice at 7,200 rpm for 30 s, pausing for 10 s between each, using Bertin Precellys Evolution Super Homogeniser, with liquid nitrogen supplied by Cryolys Cooling System. The lysate was extracted using reagents required for RNA extraction, such as chloroform, isopropanol, and 75% ethanol, according to the RNA extraction protocol. Nucleic acid concentration was checked and its quality verified by Thermo Scientific NanoDrop 2000 Spectrophotometer.

### Reverse Transcription-PCR and RT-qPCR

Total extracted RNA was synthesized into first strand DNA by HiScript II Reverse Transcriptase Kit (Vazyme) according to the manufacturer's protocol. PCR was performed as follows: 25°C for 10 min, 50°C for 30 min, 85°C for 5 min, and then cooled to 4°C. Real-time quantitative PCR (RT-qPCR) was carried out using Hieff® qPCR SYBR® Green Master Mix (YEASEN), according to the manufacturer's instruction, in a LightCycler 480, 384-well Real-Time PCR Detection System, Roche. The primer sequences used for RT-qPCR are shown in the Supplementary Table 2.

### Immunohistochemistry

Immunohistochemistry was carried out on 4-μm sections from paraffin-embedded osteosarcoma tissues using slicer system (Ventana Discovery XT automated system). In short, tissue slides were dewaxed in xylene, hydrated using gradient ethanol and then subjected to heat induced epitope retrieval. Goat serum (ZSGB-BIO, Beijing) was using for non-specific antigen blocking. The sections were incubated with primary antibody CD4 (1:100, ZSGB-BIO, #ZA-0519), CD68 (1:100, ZSGB-BIO, #ZM-0060), IFNGR2 (1:50, Sigma, #A104903), or CSF1R (1:400, Abcam, #ab215441) overnight at 4°C. Concrete details of primary antibodies used in this research is listed below in Supplementary Table 3. After incubating with secondary antibodies (ZSGB-BIO, Beijing), sections were stained with DAB and counterstained with hematoxylin. The final immunohistochemistry scoring were evaluated and recorded by two independent pathologists without knowing patients' relevant information. The numbers of CD4+, CD68+ cells were counted in five high power fields (400X HPF) in microscopes randomly. The density staining scores of IFNGR2+, CSF1R+ cells was defined as previously reported (32): none of cells stained: 0 point, <5% of cells stained:1 point, 5–50% of cells stained: 2 point or >50% of cells stained: 3 point. The intensity of staining was evaluated as 0, 1, 2, or 3 point based on negative, weak, intermediate, or strong staining, respectively, and the final scores was calculated as the multiplication of the density and intensity scores. The mean values of scores above were calculated for analysis.

### Statistical Analyses

All statistical analyses and data visualization were performed in R v3.5.2 and Prism v5 (GraphPad). To compare gene expression data from RNA-Seq expression and RT-qPCR results, we calculated Spearman rank correlations of gene expression for all possible gene pairs across the samples using the function *cor* in R or with Prism. We used one-way analysis of variance (ANOVA) or Student's *t*-test to measure statistical significance of the calculated results. For the above comparisons, *p* < 0.050 was considered statistically significant. All data are presented as mean ± SD; ^*^p < 0.050 and ^**^p < 0.010. Statistical differences were calculated by two-tailed *t*-test along with Mann-Whitney *U*-test between two groups.

## Results

### Hallmark Gene Set Enrichment and Immune Cell Infiltration Analysis for OSA

**Figure 1:**
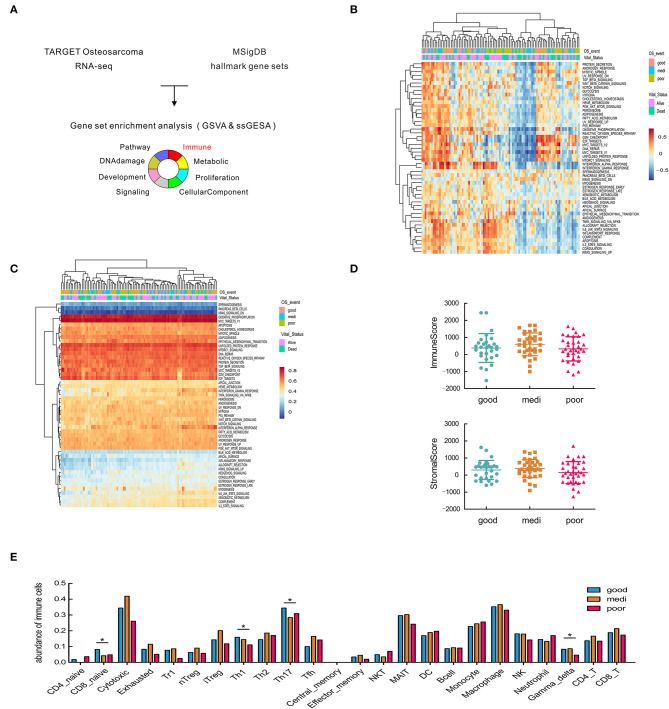
Significant heterogeneity in immune-related gene sets among patients with osteosarcoma and its correlation with disease prognosis. **(A)** Schematic representation of gene set enrichment analysis. **(B,C)** Heatmap of GSVA and ssGSEA analysis in the hallmark gene set. **(D)** Estimate of overall immune and stromal scores based on gene expression data. **(E)** Comparisons of 24 tumor-infiltrating immune cells among groups. Asterisks (*) indicate results that are statistically significance at *p*-value. *p < 0.05; **p < 0.01.

As objective responses of immunotherapy manifest in a fraction of OSA patients (4, 33), we hypothesized that immune protective signature genes may be dysregulated, which would in turn affect tumor progression and clinical responses. To confirm this hypothesis, a comprehensive study of transcriptional level information from OSA patients was carried out. We downloaded the Hallmark gene set, which is classified into eight categories (34), from MSigDB as the reference to perform gene-set enrichment analysis (Figure 1A); the analysis was performed by two different unsupervised methods of GSVA and ssGSEA (Figures 1B,C, Supplementary Tables 4, 5). Although other pathways also exhibited changes, the immune related gene-set showed heterogeneous expression in OSA patients by both analysis methods. Based on this, it was considered that deregulation of the immune pathway may create a favorable TIME for tumor progression and immune escape. Therefore, we further conducted immune infiltration analysis.

To predict and compare the overall level of immune infiltration, we calculated stromal and immune scores by the ESTIMATE algorithm (26). No significant difference was observed in overall infiltration between patients with different prognoses (Figure 1D), prompting us to consider different subsets of immune cells. Hence, ImmuCellAI was applied to estimate the abundance of 24 immune cell types in the samples, and the difference in immune cell infiltration between patient groups (Figure 1E). Traditionally, CD4+ Th1 cells are critical mediators for facilitating sustained anti-tumor responses (35). We found a higher abundance of Th1 and Th17 cells in OSA patients with favorable prognoses, as expected (Figure 1E), while Th2 cells exhibited the opposite effect in OSA.

In contrast, macrophages were the most abundant in the microenvironment (Figure 1E), however, were not significantly different between the patient groups. Therefore, we next analyzed different macrophages paradigms and their correlation with OSA prognosis.

### Th1 Cells and M1 Macrophage Infiltration Is Associated With Favorable Prognoses in OSA

Since our analysis revealed differences in abundance of certain immune cell subsets, we selected a series of specific markers for various cell subtypes for further validation. The cell subsets were distinguished from each other based on the following markers: (a) T cell-associated CD4, CD8A, and CD8B; (b) Th1-associated IFNGR2, CCR5, and IL12RB2; (c) Th2-associated CCR3, CCR4, and IL4; (d) Th17-associated IL17A, IL1R1, and IL23R. Moreover, we also investigated the expression of T cell immune checkpoints CTLA4, PDCD1(PD-1), CD274(PD-L1), TIGIT, HAVCR2(TIM-3), and LAG3.

**Figure 2:**
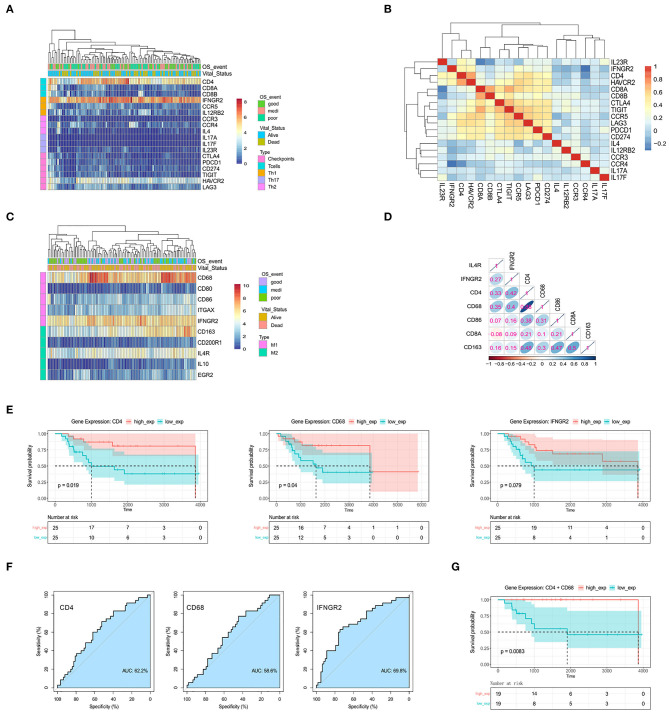
The genes CD4, CD68, and IFNGR2 encode tumor-infiltrating immune cell markers, and their expression predicts survival outcomes for patients. **(A)** Heatmap for the expression levels of T cell-related genes. **(B)** Spearman's rank correlation in T cell-related genes based on gene expression data. **(C)** Heatmap for the expression levels of macrophage-related genes. **(D)** Spearman's rank correlation in immune-related genes. **(E)** Kaplan-Meier survival for OSA patients further separated into high- and low-expression groups for CD4, CD68, and IFNGR2. **(F)** ROC curves. **(G)** Kaplan-Meier survival for CD4+CD68 co-expression groups.

Notably, the abundance of CD8+ or CD4+ T cells subsets differed consistent with the low-level expression of CD8, the expression level of CD8+ T cell-relevant immune checkpoints (e.g., CTLA4, PDCD1) was weak (Figures 2A,B). The heterogeneity observed in CD4+ Th1 related genes (e.g., IFNGR2, HAVCR2) was higher in some OSA patients; suggesting that not all T cells subsets are activated in OSA. Subsequently, we focused on CD4+ Th1 cells, which showed high abundance and heterogeneity (Figure 2A, Supplementary Table 6). To further confirm the importance of Th1 cells, we applying Cox regression to the expression of Th1 cells marker CD4 and IFNGR2. In this multivariate Cox analysis (data not shown), the covariates CD4 and IFNGR2 fails to be significant, but we found that the covariate CD4 (coef = −0.1799) and IFNGR2 (coef = −0.6451) have a negative coefficient, means lower expression of CD4 and IFNGR2 are associated with poorer survival (global *p* = 0.0339).

Some patients displayed activated M1 macrophages, characterized by increased expression of CD68 and IFNGR2 (Figure 2C), while others demonstrated higher expression of M2-type markers, CD163, and IL4R. To assess the correlation between these markers and OSA prognosis, we carried out Spearman's correlation analysis using normalized gene expression matrices (Figure 2D, Supplementary Table 7). Interestingly, the expression of the M1-marker, CD68, showed a strong correlation with CD4. We hypothesized that infiltration of activated Th1 cells and M1 macrophages may be associated with favorable prognosis in patients with OSA. We, therefore, tested Kaplan-Meier curves for the overall survival of patients with OSA according to the low and high expression of Th1 cells and M1 macrophages related genes (Figure 2E). The high expressions of CD4 and CD68 were both related to favorable prognosis of OSA (*p* < 0.05). Although no significant difference (*p* > 0.05) was observed for IFNGR2 expression, a trend was observed for poor survival of patients with low IFNGR2 expression. The AUC value of ROC evaluation was clearly not ideal considering a single factor, indicating that OSA prognosis may be affected by multiple factors (Figure 2F). We further evaluated the combined effect of both CD4 and CD68 (Figure 2G) and found that high co-expression was significantly correlated with improved survival (*p* < 0.01). Thus, the interaction between Th1 cells and M1 macrophages in OSA may serve as an essential component of immune response associated with favorable prognosis.

### Prognostic Biomarkers Associated With Immune Responses in OSA

**Figure 3:**
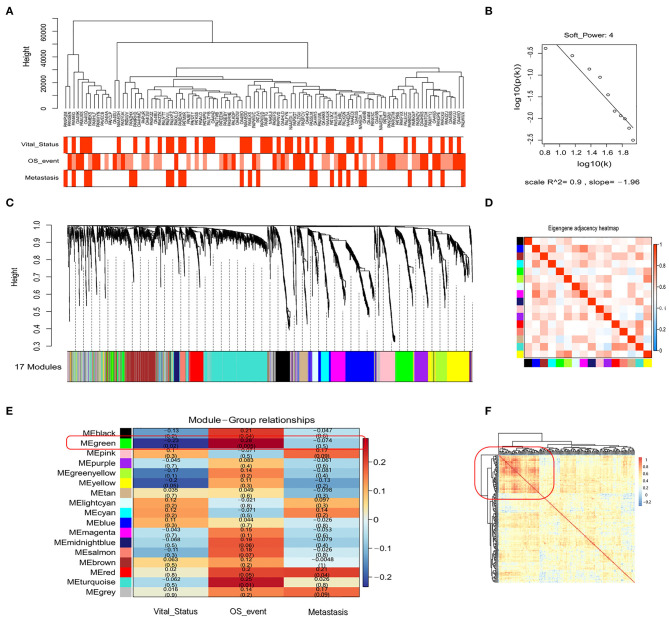
WGCNA of the genetic modules related to prognosis based on immune-related genes. **(A)** Sample dendrogram and trait indicator. **(B)** Checking the scale-free topology for the soft-thresholding power chosen. **(C)** Cluster dendrogram among patients for 17 modules by immune-related gene expression. **(D)** Heatmap of eigengene adjacency between individual modules. **(E)** Heatmap of module–trait relationships. **(F)** The clustering of genes in the green module based on Spearman's correlation coefficients between samples.

In the above analysis, we only considered recognized immune markers but did not consider other potential prognostic immune markers that may be associated with OSA. Therefore, we utilized the expression matrix comprised of 3,237 immune-related genes (GO: 0002376, immune system process) to construct a co-expression network by WGCNA. To identify the most significant modules associated with the prognosis and progression of OSA, we analyzed the association between modules and clinical phenotypes (Figure 3A). Incomplete annotations or under-expressed records were filtered out, resulting in 2,846 immune-related genes that were then grouped into modules based on similar expression patterns via average linkage hierarchical clustering. According to check scale free topology, the power of β = 4 (scale-free *R*^2^ = 0.9) was chosen for soft-thresholding to ensure a scale-free network (Figure 3B). A total of 17 modules were thus generated, and the heatmap of the correlation between module eigengenes are shown in Figures 3C,D (Supplementary Tables 8, 9). The module trait relationship is shown in Figure 3E. Most of the correlations were low to moderate (*R*^2^ < 0.5), with the green module being higher than any other module. Next, we screened out the green module that was significantly positive correlated with the Overall_Survival_event (*r* = 0.28, *p* = 0.0049), and was negatively correlated with Vital_Status (*r* = −0·23, *p* = 0.020), which was chosen for further analysis. The green module contained 209 immune-related genes, including CD4 and CD68, which were then subjected to correlation analysis (Figure 3F). Interestingly, the heatmap indicated that there was a strong correlation between some genes. Highly connected hub genes in the green module may play important roles in the tumor processes and may work synergistically to influence prognosis. Based on co-expression analysis, the green module containing the immune genes was identified as the OSA clinically significant module, and was subjected to further analysis.

### Prognostic Value of the Immune Cell Subset CD4^+^CD68^+^CSF1R^+^ in Tumor Microenvironment in OSA

**Figure 4:**
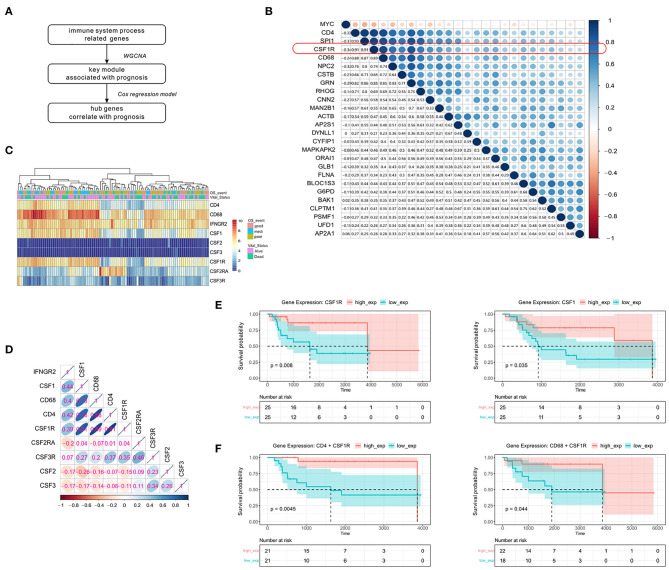
Significant correlation between the expression of immunity-related genes CSF1R, CD4, and CD68 and disease prognosis. **(A)** Simple flow chart for hub gene screening. **(B)** Spearman's rank correlation in genes screened from the Cox regression model, and CD4/CD68. **(C)** Heatmap of the expression levels of CSF family genes and immune-related genes mentioned above. **(D)** Spearman's rank correlation in genes associated with the CSF family and immune cells. **(E)** Kaplan-Meier survival of CSF1 and CSF1R. **(F)** Prognostic influence of CD4+CSF1R and CD68+CSF1R co-expression groups in Kaplan-Meier survival analysis, separately.

To identify crucial genes in the green module that might affect the overall survival of OSA patients, the univariate Cox regression model was applied to detect the prognostic genes after removing the low expression records. A flowchart of hub gene selection is shown in Figure 4A. We derived 24 genes whose expression were associated with survival time and ultimate state in the TARGET cohort (*p* < 0.01). Eventually, these 24 genes with high connectivity in green module were considered as the hub genes for tumor progression. To further elucidate the relationship between these 24 genes and immune markers that we previously screened, Spearman's correlation analysis was carried out between these genes and CD4/CD68 (Figure 4B). Compared with other genes, the expression level of CSF1R showed the strongest correlation with that of CD4 (*r* = 0.91, *p* < 0.0001) and CD68 (*r* = 0.89, *p* < 0.0001) in OSA. As CSF1R is a current hotspot in immunotherapy (36), and its role in OSA remains unclear, we further focused on CSF1R in the follow-up analysis.

To further confirm that CSF1R is indeed related to the immune markers mentioned earlier, we first examined the CSF family genes (Figures 4C,D). Only CSF1/CSF1R was found to show high expression in OSA compared to other CSF family genes, and display a strong and positive correlation with CD4/CD68. To further verify the prognostic value of CSF1R, Kaplan-Meier survival analysis based on CSF1/CSF1R expression was conducted (Figure 4E). We found that increased expression of CSF1/CSF1R in OSA patients was significantly correlated with prolonged survival. We also assessed the association between CD4 or CD68 and CSF1R co-expression and prognosis in patients (Figure 4F). The combination of either CD4 or CD68 with CSF1R predicted a more favorable prognosis (*p* < 0.01), suggesting that coordinated interaction between tumor antigen-specific CD4+ Th1 cells and CSF1R might participate in macrophage polarization toward CD68+ M1 TAM.

### CSF1R Is Associated With Altered Tumor Immune Microenvironment Characteristics

**Figure 5:**
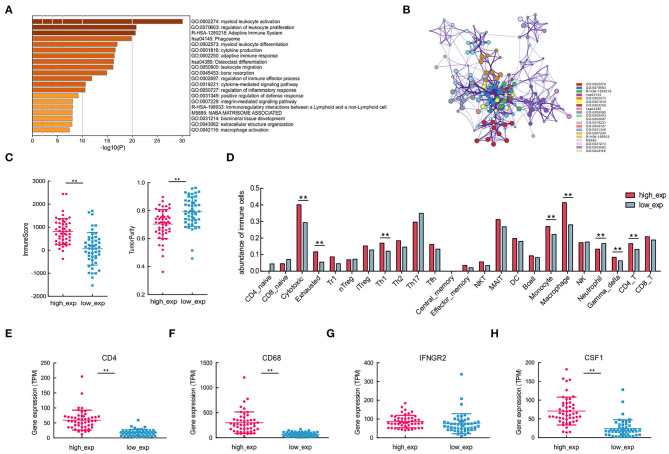
Immune cells with varying CSF1R expression patterns, associated with different molecular subtypes of CD4 and CD68. **(A,B)** Enrichment and protein-protein interaction analysis of differentially expressed genes between CSF1R high- and low-expression groups. **(C)** Comparisons of tumor-infiltrating immune cells and tumor purity depend on CSF1R expression. **(D)** Comparison of the abundance of 24 immune cells in groups with different CSF1R expression levels. **(E–H)** Comparison of the expression levels of tumor-infiltrating immune cells with gene signatures CD4, CD68, IFNGR2, and CSF1 between CSF1R high- and low-expression groups in the TARGET cohort. The *P* value: *p < 0.05; **p < 0.01.

To determine the functional role of CSF1R in OSA, patients were divided into two groups (high or low group) based on the median value of CSF1R expression using *limma*. The functional enrichment and protein-protein interaction network analyses further revealed DEGs enriched in many immune-related biological processes (Figures 5A,B). In particular, myeloid leukocyte activation (GO: 0002274, *p* < 0.01) showed the highest enrichment degree; as myeloid leukocytes are closely related to tumor-induced immunosuppression (37).

To assess immune infiltration in different CSF1R expressed groups, we first applied *estimate* for predicting the presence of infiltrating stromal/immune cells, and tumor purity. In CSF1R high-expressed group, higher immune scores and low tumor purity reflected the presence of immunoreactive gene expression subtypes (Figure 5C). It is suggested that CSF1R high-expressed group showed relatively high immune cell scores and may be associated with susceptibility to immune response. Next, we applied ImmuCellAI to examine the abundance of 24 immune cells subtypes; some of the immune cells showed differential expression pattern in the two groups (Figure 5D). As expected, CD4/CD68-related immune cell subtypes, such as macrophages, Th1, and CD4_T, were significantly different in two groups (*p* < 0.01). In addition, some immune cell subsets with anti-tumor function, such as cytotoxic and gamma-delta T cells, were actively expressed in the CSF1R high-expressed group. Further, expression analysis of CD4, CD68, and CSF1 indicated that all of them were significantly different in the two groups (*p* < 0.01; Figures 5E–H). These results suggest that the expression levels of CSF1R in OSA tumor may be associated with TIME characteristics; higher CSF1R may correlate with the abundance of CD4+ Th1 cells and CD68+ TAMs in OSA.

### CD4/IFNGR2/CD68/CSF1R Exert Synergistic Anti-tumor Effects in Response to Neoadjuvant Chemotherapy

**Figure 6:**
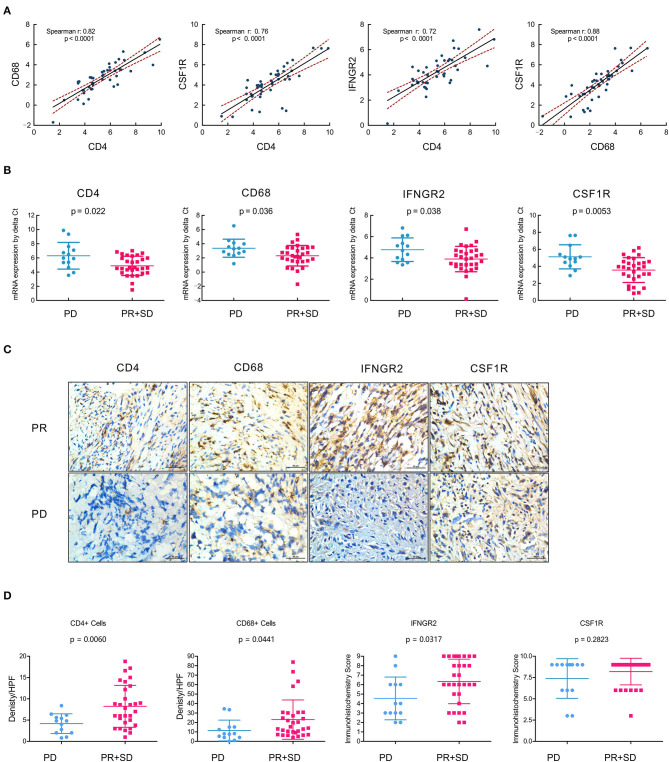
Immune contexture CD4/IFNGR2/CD68/CSF1R exerts synergistic effects and predicts prognostic neoadjuvant chemotherapy responses. **(A)** Correlation analyses of the mRNA expression levels of tumor-infiltrating immune cells with gene signatures CD4, CD68, IFNGR2, and CSF1R from the real-time quantitative PCR of OSA primary tumors (*n* = 45). **(B)** Comparison showing the mRNA expression levels of tumor-infiltrating immune cells with CD4/IFNGR2/CD68/CSF1R predicting prognostic neoadjuvant chemotherapy responses in OSA target lesions (*n* = 45); *t*-test was used to assess significance. **(C)** Immunohistochemistry for CD4/CD68/IFNGR2/CSF1R expression in the PR or PD tissues in paraffin tissue sections of osteosarcoma patients who underwent neoadjuvant chemotherapy. **(D)** Comparison of the density per HPF of CD4+/CD68+ cells and immunohistochemistry score of IFNGR2/CSF1R in non-responders (PD) and responders (PR and SD). PD, progressive disease; PR, partial response; SD, stable disease.

For further verification of the expression of CD4/IFNGR2/CD68/CSF1R as immune signatures in OSA tumor microenvironment, RT-qPCR was performed using mRNA from 45 collected primary surgical tissue samples of OSA primary tumors. As expected, we found a significant positive correlation between the mRNA expression levels of CD4, CD68, IFNGR2, and CSF1R (*p* < 0.0001; Figure 6A), with high Spearman's correlation values (CD68/CSF1R, *r*: 0.88; CD4/CD68, *r*: 0.82, and CD4/CSF1R, *r*: 0.76). Thus, an immune cell composition of CD4+CD68+CSF1R+ could exert strong anti-tumor effects with great cooperativity between these immune factors.

Next, we assessed the clinical response categories of neoadjuvant chemotherapy according to Response Evaluation Criteria in Solid Tumors (RECIST1.1) criteria through the change in tumor volume based on MRI and the development of pulmonary metastasis based on the chest CT. We divided unfavorable chemotherapy responses (PD) and favorable responses (PR+SD) as two cohorts, and found that the mRNA expression level of CD4/IFNGR2/CD68/CSF1R could predict favorable neoadjuvant chemotherapy responses in OSA target lesions (Figure 6B).

To verify the infiltration of Th1 CD4 + T cells and CD68 + macrophages plays a key role in tumor regression in OSA, immunohistochemistries of 45 post-neoadjuvant chemotherapy surgical samples were performed and found more infiltrating CD4+ T cells and CD68 + macrophages exists in the tumor center in the responders (PR + SD) compared to the non-responders (PD), indicating more infiltration of CD4+ T cells and CD68 + macrophages are relevant to a good histopathological response (Figures 6C,D). Hence, the infiltration of Th1 CD4 + T cells and CD68 + macrophages in tissues is the key to affecting the efficacy of neoadjuvant chemotherapy and prognosis in osteosarcoma patients.

## Discussion

Our study suggests that the presence of CD4+ Th1 cells and CD68+ macrophages may indicate a better prognosis in OSA. Our analysis identified the prognostic gene signature of CD4/CD68/CSF1R, and revealed the intrinsic relevance of these molecular alterations and immune features in predicting clinical outcomes and chemotherapy responses in patients with OSA.

Similar to chemo-immunotherapy or dual-immunotherapy, combination strategy promises to deliver long-term survival benefits to OSA patients that may be unavailable with current approaches. The tumor microenvironment plays an important role in sarcomagenesis and immunotherapy, hence, a growing number of studies have focused on the tumor microenvironment in OSA (1, 38). However, it remains uncertain how different immune cells associate with OSA phenotype and affect pathogenesis. In this study, transcriptome data analysis showed that tumor infiltrating cells were highly heterogeneous. In addition to analyzing the influence of CD4 and CD68, we also explored other potential prognostic immune genes. Similarly, naive CD8+ T cells can respond to tumor antigens and differentiate into cytotoxic effector cells, while γδT cells have the property of killing tumor cells (39, 40). Our analysis revealed that although naive CD8+ and γδT cells were significantly related to prognosis, they exhibited low abundance in OSA; hence, we did not carry out further in-depth analysis of these cells.

Seventeen modules were identified by WGCNA, one of which, namely CSF1R, showed the strongest correlation with prognosis. After multiple screenings, we found that the immune response signature of CSF1R was associated with improved survival in OSA, with a strong correlation observed between CSF1R and CD4/CD68 (Figures 4, 5). Based on TARGET analysis and consistent RT-qPCR data for 45 patient samples, our results strongly suggest that the new immune-related gene signature of CD4/CD68/CSF1R has prognostic value for OSA.

Infiltrated immune cells could be act as markers of the immune response, shown the correlation of response to therapy and overall survival, like higher T cells and macrophages infiltration were often believed to indicate a better prognosis (41–43). Here, we report that CD4+ Th1 and γδT cell deficiency was associated with short-term survival (Figure 1E). Notably, the abundance of these cells increased along with high expression of CSF1R (Figure 5D). Th1 cells promote anti-tumor immune responses by activating antigen-presenting cells, and the M1 form of TAM also display anti-tumor function, which is positively associated with survival (44). As CD4 and IFNGR2 together contribute to the activation of Th1 cells (45), and M1 macrophages are characterized by CD68, we were able to validate the correlation of these immune signatures (Figures 4D, 6A).

The standard treatment for OSA patients includes traditional neoadjuvant chemotherapy and surgical resection of localized tumors, followed by additional adjuvant chemotherapy according to NCCN guidelines (46). It was reported that assessable response to neoadjuvant chemotherapy was the strongest predictor of overall survival for patients with localized disease (47). Interestingly, we found that these immune-related genes (CD4/CD68/IFNGR2) show a significantly positive correlation with each other, and may indicate a more favorable clinical response (Figures 6A–D). Clinical sample results show that the infiltration abundance of CD68 +macrophages and Th1 CD4 + cells was positively correlated with each other and prone to be better prognosis of osteosarcoma and efficacy of neoadjuvant chemotherapy in OSA. As Th1 cells and macrophage infiltration were abundant and prone to be the predominant immune cell subsets in OSA immune microenvironment, the gene signature of CD4+IFNGR2+CD68 might result in synergistic anticarcinogenic effects via augmentation of phagocytosis of OSA cells.

Although our study identified this prognostic gene signature, function of these immune cell subsets, and validation of the molecular mechanisms require further investigation. Furthermore, the molecular mechanism of CSF1/CSF1R in OSA remains unclear; the positive correlation with the expression of CSF1R and CD4/CD68 is not clear that it must play the positive effect on prognosis, and it may also be as a cancer promoting factor that involved in tumor immune escape. Immunohistochemical results showed that CSF1R was expressed not only on the surface of macrophages, but also in tumor cells with high abundance (Figure 6C). Many studies this year have also suggested that CSF1R plays an important role in functional maintenance and genetic Rescue of osteoclasts (48, 49). As OSA is begins in cells that form bones, whether CSF1R have some specific role in the immune microenvironment of bone tumors. Future studies should examine whether CSF1/CSF1R acts synergistically with CD4+ Th1 cells to activate anti-tumor CD68+ macrophages, or adaptive CSF1 secretion upon exposure to CD4+ T cell-derived cytokines act detrimentally to recruit M2-like TAMs and consequently hamper antitumor immune responses.

It is likely that a minority of OSA patients may benefit greatly from additional immunotherapy (50); thus, identification of prognostic factors that indicate the subclasses of TIME may aid in patient selection for subsequent rounds of therapy. Herein we effectively observed improved prognosis in patients with strong CD4/CD68/CSF1R expression suggesting that these immune response signatures may correlate with patient diversity allowing for personalized, precision medicine, leading to OSA-tailored therapeutic strategies. However, low patient numbers is a limitation of our study, hence, our findings must be confirmed in larger cohorts.

## Data Availability Statement

The results published here are in whole or part based upon data generated by the Therapeutically Applicable Research to Generate Effective Treatments (https://ocg.cancer.gov/programs/target) initiative, phs000218. The data used for this analysis are available at https://portal.gdc.cancer.gov/projects.

## Ethics Statement

The studies involving human participants were reviewed and approved by the Medical Ethics Committee of the Sun Yat-sen University Cancer Center. Written informed consent to participate in this study was provided by the participants' legal guardian/next of kin.

## Author Contributions

JW, QT, YS, and XY designed the research. YS and YX wrote the paper, with contributions from and discussion with all of the co-authors. XZ, JF, CD, HC, HX, GS, and JL conducted the research. All authors have read and approved the manuscript.

## Conflict of Interest

The authors declare that the research was conducted in the absence of any commercial or financial relationships that could be construed as a potential conflict of interest.
