# Supplementary material for: Immune Landscape of the Tumor Microenvironment Identifies Prognostic Gene Signature CD4/CD68/CSF1R in Osteosarcoma
Source: Front Oncol. 2020 Jul 24;10:1198. doi: 10.3389/fonc.2020.01198 (PMC7396617; doi:10.3389/fonc.2020.01198)
Supplement: Supplementary file 1 [file Data_Sheet_1.zip › data sheet 1/Supplementary Tables 1- 3.doc]

## Supplement Table 1

Clinical characteristics of 47 osteosarcoma patients

| Characteristics | | Total (n=45) | Percentage（%） |
| --- | --- | --- | --- |
| Age, years (6 - 44) | ≤ 10 | 8 | 17.8 |
|  | 10 - 18 | 27 | 60.0 |
|  | 18 - 44 | 10 | 22.2 |
| Gender | Male | 24 | 53.3 |
|  | Female | 21 | 46.7 |
| Primary tumor site | Femur | 29 | 64.4 |
|  | Tibia/Fibula | 10 | 22.2 |
|  | Humerus/Radius | 5 | 11.1 |
|  | Pelvis | 1 | 2.2 |
| Enneking | IIB | 25 | 55.6 |
|  | III | 20 | 44.4 |
| Relapse | Yes | 1 | 2.2 |
|  | No | 44 | 97.8 |
| Lung metastasis | Yes | 19 | 42.2 |
|  | No | 26 | 57.8 |
| Treatment response | PR | 18 | 40.0 |
|  | SD | 12 | 26.7 |
|  | PD | 13 | 28.9 |
|  | NA | 2 | 4.4 |

Progressive disease, PD; Partial response, PR; Stable disease, SD

## Supplement Table 2

Primers used in this study are as follows:

| Gene | Forward primer(5'to3') | Reverse primer(5'to3') |
| --- | --- | --- |
| CD4 | TGCCTCAGTATGCTGGCTCT | GAGACCTTTGCCTCCTTGTTC |
| CD68 | GGAAATGCCACGGTTCATCCA | TGGGGTTCAGTACAGAGATGC |
| IFNGR2 | CTCCTCAGCACCCGAAGATTC | GCCGTGAACCATTTACTGTCG |
| CSF1R | GGGAATCCCAGTGATAGAGCC | TTGGAAGGTAGCGTTGTTGGT |
| GADPH | TTCTTTTGCGTCGCCAGCCGA | GTGACCAGGCGCCCAATACGA |

## Supplement Table 3

The details of primary antibodies used in this research.

| **Antigen** | **Product Numbers** | **Supplier** | **Species** | **Dilution** |
| --- | --- | --- | --- | --- |
| CD4 | ZA-0519 | ZSGB-BIO | Rabbit | 1:100 |
| CD68 | ZM-0060 | ZSGB-BIO | Rabbit | 1:100 |
| IFNGR2 | A104903 | SIGMA | Rabbit | 1:50 |
| CSF1R | ab215441 | ABCAM | Rabbit | 1:400 |
